# Framing poor diet quality as malnutrition: the *Brazilian National Survey on Child Nutrition* (ENANI-2019)

**DOI:** 10.1590/0102-311XEN089222

**Published:** 2023-09-25

**Authors:** Gyorgy Scrinis, Inês Rugani Ribeiro de Castro

**Affiliations:** 1 School of Agriculture and Food, University of Melbourne, Melbourne, Australia.; 2 Instituto de Nutrição, Universidade do Estado do Rio de Janeiro, Rio de Janeiro, Brasil.

**Keywords:** Malnutrition, Nutritional Transition, Obesity, Micronutrients, Preschool Child, Desnutrição, Transição Nutricional, Obesidade, Micronutrientes, Pré-escolar, Desnutrición, Transición Nutricional, Obesidad, Micronutrientes, Preescolar

## Abstract

Based on the *Brazilian National Survey on Child Nutrition* (ENANI-2019) results, this article reflects on the adequacy of the “malnutrition in all its forms” framework and system of classification for representing and interpreting these dietary transitions in Brazilian children. We highlight the limitations of this classification system, including the focus on health outcomes and anthropometric measures, the siloed understanding of these forms of malnutrition, the lack of relevance of the obesity category to children under 5 years old, and the failure to adequately address the various measures of poor quality diets captured by ENANI-2019. As an alternative, based on an approach developed by Gyorgy Scrinis to reframing malnutrition in all its forms, we suggest a need for frameworks that focus on describing and classifying the nature of, and changes to, dietary patterns, rather than focused on health outcomes.

## Abstract (pt)

Com base nos resultados do *Estudo Nacional de Alimentação e Nutrição Infantil* (ENANI-2019), este artigo reflete sobre a adequação da estrutura e do sistema de classificação “má nutrição em todas as suas formas” para representar e interpretar essas transições alimentares em crianças brasileiras. Este estudo destaca as limitações desse sistema de classificação, incluindo o foco nos resultados de saúde e medidas antropométricas, o entendimento isolado dessas formas de má nutrição, a falta de relevância da categoria obesidade para crianças menores de 5 anos de idade e a incapacidade de abordar adequadamente as várias medidas de dietas de baixa qualidade identificadas pelo ENANI-2019. Como alternativa, com base em uma abordagem desenvolvida por Gyorgy Scrinis para reformular a abordagem da má nutrição em todas as suas formas, são necessárias estruturas que se concentrem em descrever e classificar a natureza e as mudanças nos padrões alimentares em vez de se concentrar nos resultados de saúde.

## Abstract (es)

Con base en los resultados del *Estudio Nacional de Alimentación y Nutrición Infantil* (ENANI-2019), este artículo reflexiona sobre la adecuación del marco y del sistema de clasificación de la “malnutrición en todas sus formas” para representar e interpretar estas transiciones dietéticas en los niños brasileños. Este estudio destaca las limitaciones de este sistema de clasificación, incluido el enfoque en los resultados de salud y las medidas antropométricas, la comprensión aislada de estas formas de malnutrición, la falta de relevancia de la categoría de obesidad para niños menores de 5 años y la incapacidad de abordar adecuadamente las diversas medidas de dietas de baja calidad identificadas por el ENANI-2019. Una alternativa sería, basándose en un enfoque desarrollado por Gyorgy Scrinis para reformular el enfoque de la malnutrición en todas sus formas, establecer estructuras que se centraran en describir y clasificar la naturaleza y los cambios en los patrones dietéticos en lugar de centrarse en los resultados de salud.

## Introduction

Recently, middle-income countries such as Brazil have been undergoing rapid dietary transformations, closely followed by changes in the types and patterns of diet-related health issues ^1^ ^,^ ^2^ ^,^ ^3^. The ways in which we categorize and track these dietary changes and their associated health impacts is important for they frame both our understanding of these issues and how we respond to them. But there are limitations associated with some of the standard frameworks for representing these transformations, particularly for children in these countries.

Dietary transformations in low- and middle-income countries such as Brazil are commonly described in terms of the “nutrition transition” concept, developed by Barry Popkin to characterize the secular trends in nutrition patterns resulting from changes in food intake as a consequence of economic, social, demographic, and health changes ^4^ ^,^ ^5^. The nutrition transition framework largely describes the dietary dimension of this transition in general terms, from traditional foods to high-protein and highly-processed diets, and diets with high sugar, salt, and fat content. The nutrition transition now also acknowledges that the nature and pace of changes in dietary patterns can vary between places, but that the broad effects on nutritional status and health are similar ^5^.

Yet, the notion of transition from traditional eating habits to other habits may be an over-generalization of the reality of the multiple transitions, transformations, and pathways that different countries and communities are experiencing. There are other previous frameworks that provide ways of describing and analyzing the types of dietary transformations experienced worldwide, particularly frameworks that provide ways of measuring and evaluating the quality of food-based rather than nutrient-based dietary patterns ^6^ ^,^ ^7^ ^,^ ^8^. When this nutrition transition framework was first developed, there were no existing or widely accepted systems for classifying types of processed foods, such as the NOVA classification ^9^.

There also are not any similar frameworks for classifying and representing transformations in the “health impacts” associated with these dietary transformations. Nutrition transition partially intends to capture the shift in forms of malnutrition, from undernutrition to the so-called “overnutrition”, obesity, and noncommunicable diseases (NCDs). The notion of “malnutrition in all its forms” has become the dominant way in which these myriad forms of malnutrition and dietary health issues are now being defined and measured ^10^. This framing has taken the form of a dualistic distinction between undernutrition and “over” nutrition, or a tripartite distinction between undernutrition, micronutrient deficiencies, and obesity.

The results of the *Brazilian National Survey on Child Nutrition* (ENANI-2019) show a mixed scenario of dietary and health transformations, with some encouraging indicators suggesting improvements in dietary health in Brazil, and others signaling the persistence of old problems and a continued rise in relatively new ones. The health indicators suggest an improvement in some conditions traditionally related to undernutrition issues (anemia and vitamin A deficiency), alongside the continued rise in excess weight ^11^. However, the dietary indicators provide a more nuanced picture of the nature of dietary patterns and the malnutrition status of children ^11^.

Based on the approach developed by Gyorgy Scrinis ^10^, this article reflects on the suitability of the “malnutrition in all its forms” framework and system of classification to represent and interpret these dietary transitions among Brazilian children. We highlight the limitations of this classification system and suggest a need for frameworks focused on describing and classifying the nature of and changes in dietary patterns, rather than focusing only on health outcomes.

## Summary of dietary and health indicators

The findings of ENANI-2019 have been extensively described in other articles in this special issue ^11^ ^,^ ^12^ ^,^ ^13^ ^,^ ^14^. Here we present a brief summary of these findings. Comparing the results of the *Brazilian National Survey of Demography and Health* (PNDS 2006) ^15^ with those of the ENANI-2019, we observed: a decrease in the prevalence of anemia (hemoglobin < 11g/dL) and vitamin A deficiency (serum retinol < 0.7µmol/L) from 20.5% to 10.1% and of 17.2% to 6%, respectively ^11^; stability of the prevalence of stunting [(height-for-age Z-scores < -2, based on the World Health Organization (WHO) growth curves] at 7%, but with an indication that older children are better off than younger children ^14^; an increase in the prevalence of indicators of thinness (body mass index - BMI-for-age Z-scores < -2, based on WHO growth curves) (from 1.7% to 3%) and of low weight for age (weight-for-age Z-scores < -2, based on WHO growth curves) (from 1.9% to 2.9%) ^14^ (with no statistical difference); and an increase in excess weight (BMI-for-age Z-scores > 2, based on WHO growth curves) (from 6.6% to 10.1%) ^11^. An increase in the prevalence of breastfeeding indicators was also observed (with no statistical difference): exclusive breastfeeding (up to 5.9 months; from 38.6% to 45.8%); and continued breastfeeding (12-23.9 months; from 34.6% to 46.3%) ^16^.

Overall, considering the geographic regions of the country, the maternal education levels and maternal race/skin color, the socio-demographic disparities in the prevalence of anemia, vitamin A deficiency, stunting, and breastfeeding decreased from 2006 to 2019. On the other hand, disparities between geographic regions increased for excess weight in this same period ^11^.

Considering the food that children 6-59 months of age had consumed on the day before the ENANI-2019 interview, 55.6% reached the minimum dietary diversity recommended by the WHO ^17^; 25.7% had not consumed either fruits or vegetables; and 89% had consumed ultra-processed foods, with 43% having consumed three or more types of ultra-processed foods ^12^ ^,^ ^18^.

## Limitations of the standard tripartite classification of malnutrition

The dominant approaches to represent, measure, and analyze malnutrition are the dualistic and the tripartite classifications, which define and divide malnutrition into either two categories - undernutrition versus overnutrition/obesity; or three categories - chronic undernutrition, micronutrient deficiencies, and obesity/overweight. For example, WHO distinguishes between the three categories of undernutrition, micronutrient-related malnutrition, and overweight/obesity/diet-related noncommunicable diseases ^19^.

This classification emerged in the late 1990s from the attempt to expand the earlier focus on malnutrition as undernutrition/hunger ^20^. Prior to this, the dietary determinants of undernutrition were largely defined and explained by an absolute lack of food, and measured in terms of energy intake. Thus, the new categories of micronutrient deficiencies and obesity were added to capture the growing recognition that poor-quality diets produce and exacerbate other forms of malnutrition ^21^. This classification of malnutrition is important as it provides an overview of the types of malnutrition prevalent in a certain population and links these forms of malnutrition to their dietary patterns and to health outcomes. This classification also frames and shapes dietary and policy responses to malnutrition ^22^.

These categories of malnutrition are intended to represent and classify the “nutritional status” of a person. Malnutritional status is generally understood as a state of nutrition related to an inadequate diet, and that may then lead to other diseases or negative health outcomes ^23^ ^,^ ^24^. This intermediate condition of malnutritional status occurs temporally after consumption, but prior to the development of associated conditions; however, “nutritional status” is difficult to define and to measure. Inadequate levels of some micronutrients in the body can be directly measured in some cases ^23^; but the deficiency or excess of nutrients or energy intake cannot be directly measured by a biomarker, and instead, anthropometric measures are used to indicate stunting, underweight, and overweight status.

The way in which these forms of malnutrition have been defined suggests that there are fairly clear and direct relationships among the three temporal stages of (i) dietary intake; (ii) nutritional status; and (iii) health outcomes. This has led to a siloed understanding of these three forms of malnutrition as being separate and distinct.

Undernutrition is defined as a state of nutrient deficiency. This is generally defined in terms of an insufficient energy intake, measured in calories/kilojoules. Undernutrition also includes a state of micronutrient deficiency, although the category of micronutrient deficiencies specifically addresses this condition. Being in a state of undernutrition can lead to health outcomes such as stunting and underweight, which are defined by anthropometric measures. There is no way to directly measure the nutritional state of inadequate energy intake. Instead, the prevalence of stunting and underweight outcomes are used to measure and represent its prevalence. However, stunting in childhood has also been linked to the incidence of obesity in adulthood ^25^.

Micronutrient malnutrition typically refers to the nutritional status of being deficient in one or more micronutrients. As a standalone category of malnutrition, it can also refer to a state of sufficient energy intake, while lacking the intake of specific micronutrients. Micronutrient malnutrition relates to the common contemporary condition of consuming an adequate quantity of food but insufficient dietary quality and diversity. Micronutrient malnutrition is defined in very nutrient-specific terms. The dietary determinants of micronutrient malnutrition are defined as a dietary pattern deficient in specific micronutrients, which leads to a poor nutritional status in these specific micronutrients and, in turn, to micronutrient deficiency-related diseases, such as iron-related anemia. There are also non-dietary determinants of micronutrient deficiencies, such as unhealthy environments and inadequate child care practices ^26^, that can favor the occurrence of diseases that compromise the biological use of nutrients. Micronutrient deficiencies are generally presented as occurring in isolation, yet a poor-quality diet is likely to lead to multiple nutrient deficiencies. Moreover, micronutrient-deficient diets have also been shown to contribute to health outcomes related to both undernutrition (such as stunting) and obesity (such as NCDs) ^27^. The solutions to micronutrient malnutrition also tend to be framed in terms of single-micronutrients, such as micronutrient supplementation and fortified food, thereby ignoring the connections between poor-quality diet, multiple nutrient inadequacies, and multiple health outcomes.

The category of obesity/overweight is also sometimes referred to as “overnutrition”, although the later term is now rarely used, probably because of its problematic assertion that one can be “over-nourished”. Nevertheless, a primary dietary determinant of obesity is generally assumed to be excess of energy or nutrients-to-limit intake (excess free-sugars, sodium, fats), which are in turn associated with unbalanced, poor-quality and high in ultra-processed foods diets ^8^. However, excess energy/nutrient intake cannot be directly measured in the body. Instead, the prevalence of this form of malnutrition is measured by the incidence of overweight/obesity. In turn, obesity is considered to be a risk factor, or cause, of a range of NCDs. Disrupting this framing of obesity is the evidence linking undernutrition and micronutrient deficiencies to the incidence of obesity ^28^; and that people with ‘normal’ weight and people with underweight also develop NCDs. More importantly, people may have poor-quality eating habits, perhaps high in ultra-processed foods, but not develop obesity. 

The siloed understanding of these three forms of malnutrition implies that separate groups of people are experiencing these forms of malnutrition. Yet, there is abundant evidence to suggest that each of these forms of malnutrition is associated with multiple dietary determinants and health outcomes. The studies on the double-burden of malnutrition, for example, have stressed the interconnections between these multiple forms of malnutrition, such as those between stunting, micronutrient deficiencies, and obesity ^29^. There are also non-dietary determinants and contributors to the conditions of micronutrient conditions, stunting, obesity, etc. ^30^ ^,^ ^31^. Despite this mounting evidence of the interconnections between all forms of malnutrition, each of its forms continues to be represented and analyzed as if they were separate and distinct from each other.

Another limitation of the conventional classification and definitions of malnutrition is that they are defined in nutrient-specific terms: inadequate or excess energy intake, insufficient micronutrient intake, and excess nutrients-to-limit are the dietary exposures of concern. But these nutrient-specific definitions do not adequately address the quantity, diversity, and quality of dietary patterns that contribute to poor health and cannot be reduced to specific nutrients or nutrient profiles.

A more general limitation of this conventional classification and definition of malnutrition is that it does not directly address the incidence of inadequate and poor quality diets. Diets that may be sufficient regarding energy intake but are based on nutritionally unbalanced ultra-processed foods combined with a lack of diversity of minimally-processed foods are not well represented in this framework, other than as a form of “overnutrition” and as possible contributors to obesity and NCDs. Poor-quality diets - particularly those consisting of ultra-processed foods - are primarily measured via their influence on obesity, rather than for other types of health impacts that may be independent of obesity and body fat. High ultra-processed food intake in low-income countries has also been associated with micronutrient deficiencies and low dietary diversity ^32^.

In the case of children under 5 years old, the category of obesity/overweight is even less relevant than for adults, as children are less likely to achieve overweight or obese BMI status before this age, although the incidence of overweight has been increasing in Brazil and other countries, even in this age group ^11^ ^,^ ^33^. The association of the obesity category with NCDs is also not very relevant for children under 5 years old.

This also highlights some limits of measuring diet and health outcomes based on anthropometric measures. One of the consequences of measuring malnutritional status with anthropometry, or other diet/health outcomes, is the long lag in time between dietary intake and these health outcomes. The narrow framing of these forms of malnutrition regarding nutrient-specific determinants and health-specific outcomes, and as separate siloed conditions, also promotes the idea that each form of malnutrition can be addressed separately, through separate and nutrient-specific or technological “solutions”.

## ENANI-2019 indicators and the tripartite classification

The limitations of the tripartite classification of malnutrition in all its forms are evident if applied and used to interpret the ENANI-2019 survey data. Some of the ENANI-2019 survey parameters can be mapped onto the three forms of malnutrition, but others do not neatly or exclusively fit, or do not fit at all, into one of these categories. 

The category undernutrition consists of the anthropometric measures: low height-for-age (7%), low weight-for-age (2.9%), and thinness (3%). Although these values are low, the fact that older children present better results in the indicator height-for-age compared to younger children, and the finding of some increase in the prevalence of low weight-for-age and thinness indicators, suggests a deterioration of living conditions in recent years. Inadequate breastfeeding (54% of the children < 6 months were not exclusively breastfed) could also be understood as a type of undernutrition.

Micronutrient deficiencies - those that have been directly measured in the body - are vitamins A (6%), B12 (14.2%) and D (4.3%), Zinc (17.8%), and the prevalence of anemia (10.1%) ^11^ ^,^ ^34^ may only partly be attributed to micronutrient deficiencies in the diet. Notably, these figures include children with multiple micronutrient deficiencies. The decline in rates of anemia and vitamin A deficiency from 2006 to 2019 suggests an overall improvement in this form of malnutrition and probable improvements in diet quantity and quality over this period.

The obesity category is measured by the prevalence of excess weight (10.1%), of which 3% are obese. The increase in excess weight from 6.6% to 10.1% suggests that this form of malnutrition - and its associated dietary determinants - are becoming a more serious problem. But given the age of the children, even this increase may be underestimating the scale of this problem. Ultra-processed foods consumption is the dietary indicator most strongly linked to obesity. The fact that 89% of children had consumed ultra-processed foods, with 43% having consumed three or more types of these foods, shows that this is already a major concern and a significant contributor to poor-quality diets. Studies surveying the consumption of ultra-processed foods in Brazilian adults have demonstrated substantial increases in consumption of ultra-processed foods over this period, and this is likely to be reflected in increases in children’s consumption ^35^ ^,^ ^36^.

There are various indicators of poor-quality diets that do not easily map into the obesity category. Aside from ultra-processed foods consumption, low minimum dietary diversity (55%) and non-consumption of fruit and vegetables (25%) are also concerning. The lack of data on how these parameters have changed from 2006 to 2019 also hinders the generalization about dietary trends over this period; however, these indicators suggest that a significant proportion of children may be accessing an energy-sufficient diet but increasingly relying on cheap ultra-processed foods and a narrow range of fresh and minimally processed foods. 

Such poor-quality dietary patterns may be contributing to more than one of the three conventional categories of malnutrition, such as micronutrient deficiencies and overweight ^37^. However, it is difficult to attribute these diet-quality parameters to each form of malnutrition. High ultra-processed foods consumption may contribute to overweight but is also likely to contribute to deficiency-related conditions ^32^. The overweight/obesity category seems especially weak for addressing poor quality diets and the rise of ultra-processed food consumption in children under 5 years old. The prevalence of overweight/obesity in children under 5 years old (10.1%) may be greatly underestimating the proportion of children with a poor quality and high ultra-processed eating habits, but who are at high risk of becoming obese and developing NCDs and other health problems later in their childhood and life. 

While the ENANI-2019 provides evidence of a decline in some indicators of undernutrition/deficiency conditions, and a rise in obesity/“overnutrition” indicators, interpreting these results as a simple (and inevitable) shift from “under” to “over” nutrition may be both simplistic and misleading, and may mask some more divergent and contradictory trends in dietary patterns. 

## An alternative framing of ENANI-2019 indicators of malnutrition

The conventional categories of malnutrition are currently defined based on health outcomes and anthropometric measures. Therefore, the notion of “nutritional status” is not addressing directly whether people are consuming a nourishing diet, one that is adequate and proportionate in terms of diet quantity and quality. Malnutrition could instead be defined, classified, and measured by the type of dietary patterns. Unfortunately, there are no well-established systems for classifying different types of dietary patterns that could capture and categorize the dietary transformations or nutrition transitions taking place in Brazil and other countries.

Some of the parameters that could classify dietary patterns include those addressed in the ENANI-2019 survey: ultra-processed food consumption as well as other NOVA categories; dietary diversity; fruit & vegetable consumption; and breastfeeding. One clear trend worldwide is the emergence of dietary patterns dominated by ultra-processed foods, particularly in high-income countries such as the United States and the United Kingdom, which could be categorized as “ultra-processed dietary patterns” ^8^ ^,^ ^10^.

With the rising price of fresh foods and animal products in recent years and the successive reductions in the price of ultra-processed foods in Brazil ^38^, a dietary pattern of high ultra-processed foods and low dietary diversity and fresh foods may become more pronounced among Brazilian children in the coming years. Being able to quantify and categorize these dietary trends is important for developing policies aiming at addressing the drivers of these dietary patterns.

Beyond the re-definition and re-classification of malnutrition status, there is equally a need to develop frameworks capable of defining and measuring the multiple and varied dietary transitions and transformations occurring worldwide, both between and within countries and populations ^39^. Such frameworks could acknowledge multiple dietary patterns and pathways in terms of some of the dietary criteria identified above. It is important to be able to identify and measure these divergent dietary patterns within populations, particularly for those populations with extreme social inequalities, and to move away from a unitary understanding of a single nutrition transition that conceals otherwise divergent dietary pathways.
